# A health care professionals training needs assessment for oncology in Uganda

**DOI:** 10.1186/s12960-020-00506-7

**Published:** 2020-09-01

**Authors:** Josaphat Byamugisha, Ian G. Munabi, Aloysius G. Mubuuke, Amos D. Mwaka, Mike Kagawa, Isaac Okullo, Nixon Niyonzima, Pastan Lusiba, Peruth Ainembabazi, Caroline Kankunda, Dennis D. Muhumuza, Jackson Orem, Diana Atwine, Charles Ibingira

**Affiliations:** 1 Department of Obstetrics and Gynaecology, School of Medicine, Makerere University College of Health Sciences, Kampala, Uganda; 2 Department of Human Anatomy, School of Biomedical Sciences, Makerere University College of Health Sciences, Kampala, Uganda; 3 Department of Radiology, School of Medicine, Makerere University College of Health Sciences, Kampala, Uganda; 4 Department of Medicine, School of Medicine, Makerere University College of Health Sciences, Kampala, Uganda; 5 Department of Dentistry, School of Health Sciences, Makerere University College of Health Sciences, Kampala, Uganda; 6 Uganda Cancer Institute, Kampala, Uganda; 7 Office of the Principal, Makerere University College of Health Sciences, Kampala, Uganda; 8 Ministry of Health, Kampala, Uganda

**Keywords:** Cancer care facilities, Health care providers, Needs assessment, Sub-Saharan Africa, Training

## Abstract

**Background:**

Cancer incidence and mortality in sub-Saharan Africa are increasing and do account for significant premature death. The expertise of health care providers is critical to downstaging cancer at diagnosis and improving survival in low- and middle-income countries. We set out to determine the training needs of health care providers for a comprehensive oncology services package in selected hospitals in Uganda, in order to inform capacity development intervention to improve cancer outcomes in the East African region.

**Methods:**

This was a cross-sectional survey using the WHO Hennessey-Hicks questionnaire to identify the training needs of health workers involved in cancer care, across 22 hospitals in Uganda. Data were captured in real time using the Open Data Kit platform from which the data was exported to Stata version 15 for analysis using the Wilcoxon signed-rank test and Somers-Delta.

**Results:**

There were 199 respondent health professionals who were predominately female (146/199, 73.37%), with an average age of 38.97 years. There were 158/199 (79.40%) nurses, 24/199 (12.06%) medical doctors and 17/199 (8.54%) allied health professionals. Overall, the research and audit domain had the highest ranking for all the health workers (Somers-D = 0.60). The respondent’s level of education had a significant effect on the observed ranking (*P* value = 0.03). Most of the continuing medical education (CME) topics suggested by the participants were in the clinical task-related category.

**Conclusion:**

The “research and audit” domain was identified as the priority area for training interventions to improve oncology services in Uganda. There are opportunities for addressing the identified training needs with an expanded cancer CME programme content, peer support networks and tailored training for the individual health care provider.

## Background

According to the Globocan 2018 data, the global shares of deaths due to cancer in Africa (7.3%) was higher than the global shares of cancer incidence (5.8%). This reverse pattern of higher mortality than the incidence for Africa compared to other parts of the world is thought to be the result of differences in the distribution of cancer types and higher case fatality rates in Africa [1]. This is a continuation of previously observed global cancer trends that had shown that more than half (56.80%) of the 14.1 million cases and almost two thirds (64.90%) of the 8.2 million cancer deaths that occurred in 2012 were in the less-developed regions of the world [2, 3]. The trend in global cancer burden is projected to increase by 50% by 2030 with most of the increase occurring in the low- and middle-income countries (LMICs) [2, 4]. Currently, cancer in the African region is associated with late presentation and very high mortality [1]. A unique feature of cancers in Africa and most LMICs is the disproportionately high burden among women (56%), high proportion of infection-related cancers (30% of all cancers) and advanced-stage cancer presentation at diagnosis [1, 5–7]. One of these infection-related cancers affecting women, with high incidence and mortality in Africa, is cervical cancer, a cancer caused by high-risk Human Papilloma Virus [1, 8]. Worldwide, cancer of the cervix is the fourth most frequently diagnosed cancer and the fourth leading cause of cancer death in women with an estimated incidence of 570 000 cases and 311 000 deaths in 2018 [1]. The highest incidence rates are from Swaziland in Southern Africa while Malawi in Eastern Africa has the highest mortality rate [1]. In Uganda, cervical cancer is the most common cancer among women, followed by breast cancer.

Despite the several challenges facing cancer control and care in sub-Saharan Africa [9–15], the training of health care professionals and improvement in infrastructure for comprehensive cancer control and care have not been adopted as key interventions to improve cancer outcomes. In Uganda, it was recently reported by health care professionals themselves that many of them were not conversant with cervical cancer symptoms and signs [16]. Similar findings were reported from Kenya, where health professionals from regional and national referral hospitals reported inadequacies in training on management of patients with cervical cancer [17]. With such competency gaps among health care providers, the cancer burden is likely to remain a high health priority in Uganda and the East African region. In this study, the validated World Health Organization (WHO) Hennessey-Hicks TNA survey questionnaire was used to identify the training needs data for health professionals involved in providing oncology services [18]. The questionnaire is licenced to the World Health Organization for on-line use, as a toolkit for researchers [19], and has been used to determine training needs of health professionals in both high- and low-resource settings [20–25]. In this study, we set out to determine the training needs of health workers providing comprehensive oncology services in Uganda in a bid to improve cancer care provided to the population they serve.

## Methods

This training needs assessment (TNA) adopted a cross-sectional study design. The participating study sites were a set of public/government, private not-for-profit and private for-profit health institutions in various regions of Uganda with a known capacity for providing oncology services and care. Using these institutional inclusion criteria, 22 health facilities, as summarized in Table 1, from various parts of the country, were purposively included in the survey. The selected institutions are part of the referral network of health service delivery points for oncology under the East African Centre of Excellence for Oncology being developed with support from the East African Community.

**Table 1:** Description of the study sites

Type of study site	Number
Oncology treatment centre	2
Public national referral hospital	2
Public regional hospitals	14
Private hospitals	4

A consecutive sampling strategy was used to recruit participants until the required sample size was attained. The participants were only those health care providers who were (i) involved in direct care of cancer patients, (ii) present on site at the time of visit and (iii) provided written informed consent to participate in the survey at each of the selected study sites. The targeted sample size was obtained using the sample size calculator for proportions available from www.openepi.com [26], for the following assumptions: *α* = 0.05, *β* = 0.8, design effect of 1.2, 5% error and because we did not know how many health workers were aware of their training needs we used a hypothesized proportion of 50%. This gave a final sample size of 187 health care providers to which an additional 13 health workers were included as an allowance for loss and errors bringing the final targeted sample size to 200 health care providers.

The validated World Health Organization (WHO) Hennessey-Hicks TNA survey questionnaire was used to collect training needs data for oncology services [18]. The questionnaire is licenced to the World Health Organization for on-line use, as a toolkit for researchers [19]. The questionnaire has also been used to determine the training needs of several categories of health care professionals in both low-, middle- and high-income countries [27–31]. The survey questions were developed in line with the guidance set out in the online questionnaire manual [18]. The questionnaire comprises a list of 30 tasks that are categorized under the following domains: research, communication/teamwork, clinical tasks, administration and management. Each of these tasks is rated along a 7-point scale with respect to the importance of the task and the respondent’s job (rating A) and how well the task is currently performed (rating B). Comparisons of the rating A (for self-assessed importance) to rating B (current performance) provides an indication of the gap or training need. The greater the difference in the two ratings, the greater the training need for that particular task.

There exists an allowance in the questionnaire design for the removal of up to 25% of the original tasks (a maximum of 8) in exchange for other tasks of interest to the researcher without compromising the questionnaire psychometric properties [18]. For this study, we iteratively removed five (5) of the original items to create space for another five (5) items on various aspects of continued professional development (CPD). The tool was pilot tested on one nurse, one allied health worker and a medical doctor who each were asked whether the question items on the tool were clear to them. Random organization of the tools task items was maintained to retain the questionnaires’ integrity [28]. The final list of tasks included in the survey is provided in Table 2. These were randomly presented to the participants in two sections. The first section had the listed 30 task question items for rating. In the qualitative section of the tool, participants were asked to list up to three areas in which they felt they would benefit from further oncology training. These suggestions were entered verbatim into the data collection tool by the research assistant. Additional basic demographic information, including professional group, age and gender, was collected from each respondent. The questionnaire was digitized using the Open Data Kit (ODK) software for presentation to support real-time data collection and quality control using handheld data collection devices. A team of experienced data collection research assistants were recruited and taken through 5 days of training that orientation on the tool followed by repeated practice initially on the paper version, then later with the digital version of the questionnaire to ensure uniform understanding of all the question items and the consent process.

After obtaining informed consent, the research assistants helped capture the participants’ ratings for each of the tasks in the questionnaire. On completion, each fully filled questionnaire was immediately transmitted to a central server. The information on the server was checked in real time and notifications sent to the research team of any response that was inconsistent and needed immediate attention. The final dataset was exported as an excel sheets for cleaning, recoding and eventual analysis using STATA version 15. As has been previously described [28], we too analysed the results for the whole sample and also disaggregated the data to identify differences in the needs of different professional groups. Comparisons were made, using the median values for each group, to identify overall and specific differences for the various professional groups, at both the task and domain levels.

The Wilcoxon signed-rank test and Somers-D test [32] were used to determine the significance of the differences between the importance and related performance score of each task. Somers-D was used to test whether positive differences between the importance of a task or domain and performance of a task or domain tend to have higher values than negative differences [32]. In reporting the results of the Somers-D test, a non-parametric directional measure of the effect size, + 1 signified that all non-matching ranks had a positive training gap for the difference in the importance and performance scores of a domain or task. A value of − 1 signified that all non-matching ranks had a no need for training intervention (negative training gap), meaning that the task or domain was not important, and or they were performing satisfactorily or well, thus the higher performance score. The largest positive Somers-D value was used to identify the priority domain and top three tasks for intervention, individually for each one of the three professions and the overall study population [28]. The non-parametric testing approach as opposed to the survey tool authors’ preferred parametric approach was used due to the tools Likert scales that make it difficult to assume that the intervals in the data corresponding to perceptions from different participants were equal [28, 33]. The participants suggested topics for continuing medical education (CME) were categorized into groups corresponding to the Hensley-Hicks questionnaire tools domains of research, communication/teamwork, clinical tasks, administration and management. In all statistical tests, the level of significance was set at 0.05, and only the untransformed (asymmetric) values of Somers-D were used for the results [32]. All records with missing data were excluded from the analysis.

## Results

The study recruited 199 health care professionals from the various study sites with an average age of 39 years (SD 10.47 years, range 22 to 67 years). The majority of these respondent health care providers were female (146/199, 73.37%). There were 158/199 (79.40%) nurses, 24/199 (12.06%) medical doctors and 17/199 (8.54%) allied health professionals among the participants. There were 45/199 (22.61%) participants with a certificate, 104/199 (52.26%) with diplomas, 41/199 (20.60%) with degrees and 9/199 (4.52%) with masters and above level of education at the time of the interview. The majority of the nurse participants had either a certificate (44/158, 27.85%) or diploma (95/158, 60.13%) level of education. In comparison, the majority of the allied health workers (9/17, 52.94%) and the medical doctors (22/24, 92.67%) were degree holders.

Table 2 summarizes the ratings for the different tasks with respect to importance, and current performance for the original tools’ 25 items used in this survey. As can be observed from the table, the top three (3) tasks with the largest positive value for Somers-Delta statistic are identified as the priority task for each professional category. Accessing research resources was among the top three priority training tasks for all the health workers combined (Somers-D = 0.84 (95% CI 0.77 to 0.89)), and in the medical doctors (Somers-D = 0.82 (95% CI 0.50 to 0.94)) and nurses (Somers-D = 0.87 (95% CI 0.79 to 0.92)) sub-groups. Table 3 summarizes the training gaps for each domain according to the respondent’s profession. From this table, the research and audit domain had the highest ranking for all the health care providers combined (Somers-D = 0.60 (95% CI 0.49 to 0.69)). The research and audit domain with the largest positive value for all three sub-groupings according by profession with the exception for the allied health care providers where a tie was observed with the communication domain. The female participants were 0.92 times more likely to have larger training need gaps than the male participants (OR = 0.92, 95% CI 0.64 to 1.31, *P* value = 0.64). For every unit increase in age, the training gap reduced by 0.3% (OR = 0.997, 95% CI 0.98 to 1.01, *P* value = 0.65). These training needs gap significantly reduced with increasing level of education of the participants (OR = 0.80, 95% CI 0.66 to 0.98, *P* value = 0.02). This difference was only significant for participants with university bachelor’s degrees compared to the certificate holders (OR = 0.58, 95% CI 0.36 to 0.94, *P* value = 0.03). On average, the participants from the public institutions had a lower training needs gap than participants from private hospitals. This was not significant (coefficient − 0.02, 95% CI − 0.30 to 0.27; *P* value = 0.92).

**Table 2:** Showing the training gaps for each task by profession

Domain	Task	Professions											
		All health workers (*N* = 199)			Medical doctor (*N* = 24)			Nurses (*N* = 158)			Allied health worker (*N* = 17)		
		Importance (median)	Performance (median)	Somers-D (95% CI)	Importance (median)	Performance (median)	Somers-D (95% CI)	Importance (median)	Performance (median)	Somers-D (95% CI)	Importance (median)	Performance (median)	Somers-D (95% CI)
Communication	Establishing a relationship with patients	7	7	0.45 (0.32 to 0.56)	7	7	0.33 (− 0.06 to 0.64)	7	7	0.46 (0.31 to 0.58)	7	6	0.50 (− 0.14 to 0.85)
	Getting along with your colleagues in all disciplines	7	7	0.30 (0.10 to 0.47)	7	6.5	0.13 (− 0.15 to 0.38)	7	7	0.27 (0.03 to 0.49)	7	7	**1**
	Communicating with patients effectively	7	7	0.22 (− 0.02 to 0.44)	7	6	0.50 (0.12 to 0.75)	7	7	0.14 (− 0.13 to 0.39)	7	6	0
	Providing feedback to colleagues working in all disciplines	7	6	0.29 (0.01 to 0.53)	7	6	0	7	6	0.19 (− 0.13 to 0.47)	7	6	0.50 (− 0.12 to 0.84)
	Giving adequate information to patients and their families at all times	7	6	0.36 (0.05 to 0.61)	7	6	− 0.08 (− 0.53 to 0.41)	7	6	0.48 (0.35 to 0.59)	7	7	0
	Working as a member of an inter-disciplinary team	7	5	**0.64 (0.36 to 0.82)**	7	6	0.64 (0.20 to 0.86)	7	4	**0.61 (0.25 to 0.82)**	6	5	**1**
Administration	Inputting accurate data in written or computerized records/or routine data input	7	6	0.27 (0.03 to 0.48)	7	5.5	0.54 (0.17 to 0.77)	7	6	0.22 (− 0.06 to 0.47)	6	5	0.43 (− 0.09 to 0.76)
	Using technical equipment, including computers for everyday execution of your duties	7	5	0.48 (0.07 to 0.75)	7	5	0	7	5	0.40 (− 0.14 to 0.76)	6	5	0.63 (0.08 to 0.88)
	Undertaking administrative duties	7	5	0.55 (0.38 to 0.68)	6	5	0.72 (0.28 to 0.91)	7	5	0.50 (0.27 to 0.67)	6	5	0.80 (− 0.23 to 0.98)
Research and audit	Conducting any kind of research	7	4	0.51 (0.19 to 0.73)	7	5	0	7	3	0.53 (0.22 to 0.75)	5	4	0
	Identifying areas of practice that should be investigated	7	5	0.52 (0.31 to 0.67)	7	5.5	0.64 (0.28 to 0.84)	7	5	0.37 (0.03 to 0.64)	6	6	**0.83 (0.02 to 0.98)**
	Designing a research study	6	2	0.61 (0.17 to 0.84)	7	4	**0.94 (0.59 to 0.99)**	6.5	2	0.50 (− 0.07 to 0.83)	4	4	0.63 (0.07 to 0.89)
	Accessing research resources (e.g. time, money, information, equipment)	7	3	**0.84 (0.77 to 0.89)**	7	4	**0.82 (0.50 to 0.94)**	7	3	**0.87 (0.79 to 0.92)**	4	2	0
Management	Appraising your own performance	7	6	0.23 (− 0.06 to 0.49)	7	6	0	7	6	0.24 (− 0.10 to 0.52)	5	5	0.25 (− 0.73 to 0.89)
	Introducing new ideas at work	7	5	0.45 (0.23 to 0.62)	7	5	**0.83 (0.39 to 0.96)**	7	5	0.46 (0.24 to 0.64)	5	5	0.13 (− 0.77 to 0.85)
	Organizing your own time effectively	7	6	0.46 (0.35 to 0.56)	7	7	0.57 (0.004 to 0.86)	7	6	0.45 (0.32 to 0.55)	7	6	0.50 (− 0.14 to 0.85)
	Instructing or sharing new knowledge with colleagues and/or students about new practices or procedures	7	6	0.36 (0.17 to 0.54)	7	6	0.38 (0.05 to 0.64)	7	6	0.34 (0.10 to 0.54)	6	6	0
	Making do with limited resources	7	6	0.06 (− 0.16 to 0.27)	7	6	0.40 (− 0.17 to 0.77)	7	6	− 0.004 (− 0.24 to 0.23)	7	7	0
	Personally, coping with change in the health service	7	6	0.08 (− 0.21 to 0.36)	7	6	0.30 (− 0.05 to 0.59)	7	6	0.04 (− 0.31 to 0.31)	6	5	0.83 (− 0.07 to 0.99)
Clinical tasks	Treatment of patients	7	6	0.34 (0.23 to 0.43)	7	6	0	7	7	0.32 (0.21 to 0.43)	7	5	0
	Accessing relevant literature for your clinical work	7	5	**0.62 (0.45 to 0.75)**	7	6	0	7	5	**0.61 (0.41 to 0.75)**	6	4	0.67 (0.15 to 0.90)
	Planning and organizing an individual patient’s care	7	6	0.45 (0.34 to 0.54)	7	6	0	7	6	0.45 (0.33 to 0.55)	7	6	0
	Evaluating patients psychological and social needs	7	5	0.49 (0.22 to 0.69)	7	5	0	7	5	0.47 (0.19 to 0.68)	7	5	0
	Undertaking health promotion activities	7	5	0.60 (0.43 to 0.74)	7	6	0	7	5	0.58 (0.38 to 0.73)	6	5	0.71 (0.04 to 0.94)
	Assessing patients’ clinical needs	7	6	0.22 (− 0.13 to 0.52)	7	6.5	0.33 (− 0.06 to 0.64)	7	6	0.10 (− 0.39 to 0.55)	7	6	0

The table provides a summary of the responses to each of the tasks in the survey questionnaire by each participating group of health professions. For each task, a training gap was derived using the guide that accompanies the original validated World Health Organization (WHO) Hennessey-Hicks training needs assessment survey questionnaire source document. The items in bold represent the highest three values for Somers Delta in each health worker category

**Table 3:** Summarizing training gap by domain with respect to respondent’s profession

Domain	Professions											
	All health workers (*N* = 199)			Medical doctor (*N* = 24)			Nurse or midwife (*N* = 158)			Allied health worker (*N* = 17)		
	Importance (median)	Performance (median)	Somers-D (95% CI)	Importance (median)	Performance (median)	Somers-D (95% CI)	Importance (median)	Performance (median)	Somers-D (95% CI)	Importance (median)	Performance (median)	Somers-D (95% CI)
Communication	7	6	0.43 (0.32 to 0.53)	7	6	0.44 (0.12 to 0.67)	7	6	0.40 (0.27 to 0.51)	7	6	**0.71 (0.46 to 0.85)**
Administration	7	5	0.44 (0.29 to 0.56)	7	5	0.64 (0.46 to 0.77)	7	5	0.38 (0.20 to 0.54)	6	5	0.60 (0.32 to 0.78)
Research and audit	7	5	**0.60 (0.49 to 0.69)**	7	5	**0.75 (0.63 to 0.83)**	7	5	**0.54 (0.40 to 0.67)**	6	5	**0.71 (0.51 to 0.83)**
Management	7	6	0.21 (0.11 to 0.32)	7	6	0.48 (0.28 to 0.64)	7	6	0.16 (0.04 to 0.28)	6	6	0.42 (0.02 to 0.70)
Clinical tasks	7	6	0.46 (0.37 to 0.54)	7	6	0.56 (0.43 to 0.67)	7	6	0.45 (0.35 to 0.54)	7	5	0.61 (0.40 to 0.75)

The table provides a summary of the identified training gaps for each group of health professions participating in the study. These training gaps were identified using the validated World Health Organization (WHO) Hennessey-Hicks training needs assessment survey questionnaire. The items in bold represent the highest values for Sommers Delta for each collumn or health worker category

Table 4 provides a summary of the training suggestions from qualitative section of the tool. There were 552 suggested topics for training from 196 responding health workers that were categorized as follows: clinical tasks 461/552 (85.51%), research 34/552 (6.16%), management 30/552 (5.43%), communication 12/552 (2.17%), administration 10/552 (1.81%) and CME/CPD 5/552 (0.91%). Most of the suggestions (450/552, 81.52%) in Table 4 are from the participants who were nurses by designation. There were no differences in the categorization of the suggestions by gender (chi-square = 1.28, *P* value = 0.25), age (OR = 0.99, 95% CI 0.97 to 1.02) or level of education (OR = 0.88, 95% CI 0.63 to 1.25, *P* value = 0.48) of the participants.

**Table 4:** Participants suggested topics for CME/CPD

Category	Number (%)	Suggested topics for CME
Clinical tasks	461 (85.51%)	• Administration of chemotherapy • Cancer care training • Cancer management • Cancer drug administering • Cancer in the reproductive system in women • Cancer patient handling • Cancer screening • Care of terminal ill patients • Counselling and guidance
Research	34 (6.16%)	• Accessing clinical literature • Conducting cancer-related research • How to carry out research • Nurse research • Research in pain management • Research on new cancer drugs
Management	30 (5.43%)	• Computer basic training on data entry • Human resource constant training • Information system • Management and administration
Communication	12 (2.17%)	• Communication skills • Communication with patients and their families • Effective communication and meeting patient’s expectations
Administration	10 (1.81%)	• Administration and management • Data keeping • Data management and records-related courses
CME/CPD	5 (0.91%)	• Teaching/tutorship • Trainings in software applications

The table contains a summary of the coded items from the participants’ suggested CME topics. These topics were obtained from the open-ended questions asking participants to suggest possible oncology-related topics of interest to them for CME

## Discussion

This study set out to determine the training needs of health care providers involved in providing oncology services in Uganda. As shown in Table 3, the domain for research and audit had the largest training gap both overall and in each of the sub-groupings by profession. In this domain, accessing research resources was the task with the largest training need across all categories of health care providers in this survey (see Table 2). The other related tasks in the research and audit domain, with equally large training needs, were designing a research study, conducting any kind of research and the use of technology like computers. Each of the above tasks in the research and audit domain provides critical support for learning community of practice. Such communities of practice are composed of individuals who are actively learning and responding appropriately to the challenges related to their practice, in this case, the care of people with cancer. Learning organizations and or care systems are especially important for the LMICs like Uganda where the need to optimize available scarce resources and or innovate in the absence of recommended solutions is high. From our own experience, the re-organization of the Department of Obstetrics and Gynaecology at Mulago Hospital into subspecialties that include gynaecological oncology quickly unearthed a previously unnoticed cancer of the cervix high burden of disease that moved from 30% of all patients to 80% contribution to the gynaecological malignancies. This reorganization led to a change in care practices for these patients and increased advocacy for research, training, treatment and prevention. Such change within the context of a supportive working environment may probably foster the adoption of desired behaviours that include better communication to patients and use of research and audit skills to enhance cancer care.

From our previous published observations, it was noted that some of the health care providers are not conversant with cancer symptoms and signs [16]. This is especially true for the lower carder/level health care providers who many times did not suspect cancer and were instead providing treatments for other ailments [34]. Similar observations have been made in Kenya, where health professionals from regional and national referral hospitals reported inadequacies in training on management of patients in particular for cervical cancer [17]. There is evidence from the United Kingdom (UK) that inability to recognize the seriousness of symptoms or attributing symptoms to other more common conditions could delay appropriate help-seeking behaviour for symptoms of cancers [35, 36]. This fact is corroborated by the findings that prompt recognition of potential cancer symptoms is associated with earlier diagnosis of breast, colorectal and lung cancers in the United Kingdom [37]. As can be observed in Table 3, the clinical tasks for nurses, who in Uganda are predominantly non-degree-holding health care providers, had higher and positive Somers-D values for various tasks in this domain. This larger training need for the clinical tasks among nurses may be due to the abovementioned differences in the levels of education compared to the other health care providers. This is supported by significant association between level of education the training gap for the participants, more so for comparisons between certificate and degree holders (*P* value = 0.03). The current efforts to increase the number of degree-level trained nurses in the region may over time change the role of the health care providers’ level of education on the training needs for this aspect of the study [38]. In the meantime, efforts aimed at equipping these nursing professionals who are already providing services with appropriate knowledge and skills following an agreed upon set of nursing oncology competencies or using already developed educational resources from other countries should be encouraged. The already existing CME/CPD sessions that are discussed in the next paragraph can be leveraged to strengthen the practices of the certificate nursing professionals for improved cancer care.

In Malaysia, training of health care providers was shown to significantly lead to downstaging of cancers and hence improved survival [39]. Training of health care providers in the early detection of cancer symptoms in combination with other interventions like increased public awareness campaigns [40–42] may improve the outcomes of cancer in the East African region. Training of health care providers may create increased awareness of risk factors, symptoms and modalities of treatment for the various cancer sites and may potentially lead to prompt detection of cancer symptoms, early referral and treatment respectively. The observed differences in the training needs identified using the WHO Hennessey-Hicks tools and the suggestions for additional training may also be used as an important indicator of what is available to especially the nurse health care providers involved in cancer care. We think that the suggestions for cancer care CME/CPD sessions are the product of the health care providers familiarity and past experiences with various CME/CPD providers, most of whose oncology care-related content focuses on clinical tasks (see Table 4). If true, then expanding the content in the CME/CPD sessions could potentially lead to a quick increase in the knowledge base of health care providers on various oncology-related areas of practice without the work disruptions associated with longer forms of training interventions. The effect of increased content may be enhanced by encouraging the development of local peer support networks of respected health care provider cancer champions within the hospitals. Such networks have the potential to quickly enrich the quality and content of cancer care-related discourse and practice in these health care settings and thus improve the quality of cancer care in remote rural settings where the majority of the population resides.

Interventions to improve the quality of cancer care by frontline health care providers need to be tailored to the training needs of each individual. The Hennessey-Hicks tool, which was used in this study, can identify individual-level training needs by design. Future efforts to address the training needs identified in this survey may require additional modification to specific training interventions for each health care provider [43]. The other limitation of this study was the lower number of medical doctors and allied health workers that participated in the study. This may have arisen from the current staffing norms of the participating institutions and the short time frame for the study. While the use of the non-parametric statistical strategy for this study provides with fairly robust results, future studies may need to consider alternative recruitment strategies for comparisons across health care provider groupings. Despite these limitations, the findings summarized in this study provide an important and representative overview of the health care provider cancer training needs in this setting. Such an overview is important for the planning of targeted group-level interventions [44] that may be further tailored to address the individual needs of each frontline cancer health care provider.

## Conclusions

Research and audit was identified as the priority domain for training-related interventions to improve oncology services in Uganda. There are opportunities for addressing the identified training needs with an expanded cancer CME programme content, peer support networks and tailored training for the individual health care provider.

## Data Availability

The datasets used during the analysis for this training needs assessment are available from the corresponding author on reasonable request.

## Abbreviations

CME: Continuing medical education
CPD: Continued professional development
LMIC: Low- and middle-income country
ODK: Open Data Kit
TNA: Training needs assessment
UCIREC: Uganda Cancer Institute Research and Ethics Committee
UK: United Kingdom
UNCST: Uganda National Council for Science and Technology
WHO: World Health Organization
